# Seasonality and determinants of child growth velocity and growth deficit in rural southwest Ethiopia

**DOI:** 10.1186/s12887-018-0986-1

**Published:** 2018-02-01

**Authors:** Netsanet Fentahun, Tefera Belachew, Jennifer Coates, Carl Lachat

**Affiliations:** 1 Department of Health Education and Behavioral Sciences, College of Health Sciences, Jimma University, Jimma, Ethiopia; 2 Department of Population and Family Health, College of Health Sciences, Jimma University, Jimma, Ethiopia; 3 Friedman School of Nutrition Science and Policy, Feinstein International Center at Tufts University, Boston, USA; 4 Department of Food Safety and Food Quality, Faculty of Bioscience Engineering, Ghent University, Ghent, Belgium; 5 School of Public Health Engineering, Bahir Dar University, Bahir Dar, Ethiopia

**Keywords:** Seasonality, Growth velocity, Growth deficit, Rural Ethiopia

## Abstract

**Background:**

Ethiopia faces cyclic food insecurity that alternates between pre- and post- harvest seasons. Whether seasonal variation in access to food is associated with child growth has not been assessed empirically. Understanding seasonality of child growth velocity and growth deficit helps to improve efforts to track population interventions against malnutrition. The aim of this study was assess child growth velocity, growth deficit, and their determinants in rural southwest Ethiopia.

**Method:**

Data were obtained from four rounds of a longitudinal household survey conducted in ten districts in Oromiya Region and Southern Nations, Nationality and Peoples Region of Ethiopia, in which 1200 households were selected using multi-stage cluster sampling. Households with a child under 5 years were included in the present analyses (round 1 *n* = 579, round 2 *n* = 674, round 3 *n* = 674 and round 4 *n* = 680). The hierarchical nature of the data was taken into account during the statistical analyses by fitting a linear mixed effects model. A restricted maximum likelihood estimation method was employed in the analyses.

**Result:**

Compared to the post-harvest season, a higher length and weight velocity were observed in pre-harvest season with an average difference of 6.4 cm/year and 0.6 kg/year compared to the post-harvest season. The mean height of children in post-harvest seasons was 5.7 cm below the WHO median reference height. The mean height of children increased an additional 3.3 cm [95% CI (2.94, 3.73)] per year in pre-harvest season compared to the post-harvest season. Similarly, the mean weight of children increased 1.0 kg [95% CI (0.91, 1.11)] per year more in the pre-harvest season compared to the post-harvest season. Children who had a low dietary diversity and were born during the lean season in both seasons had a higher linear growth deficit. Being member of a highly food insecure household was negatively associated with higher weight gain. Having experienced no illness during the previous 2 weeks was positively associated with linear growth and weight gain.

**Conclusion:**

Child growth velocities and child growth deficits were higher in the pre-harvest season and post- harvest season respectively. Low dietary diversity and being part of a highly food insecure household were significantly risk factors for decreased linear growth and weight gain respectively.

## Background

Due to seasonal variability of food production, dietary intake, food security and morbidity in the developing world, many children suffer from impaired linear growth [1, 2]. Populations in low- and middle-income countries are vulnerable to seasonal food shortages due to rain-fed subsistence farming. Seasonality of food access affects millions of the world’s poor communities and contributes to some of the most widespread diseases [3].

In sub-Saharan Africa, more than 95% of farmed lands rely on low input and low output rain-fed agriculture. This results in seasonal food insecurity and malnutrition among a great number of poor families [4]. Low use of agricultural technology and poor market access contributes to seasonal fluctuations of household food consumption in particular in the more isolated rural households [5].

Climate change represents a major threat to the coming decades, particularly in Africa, which has more climate-sensitive economies than any other continent in the world. Climate change is expected to increase the burden of under-nutrition in particular in rural households [6, 7]. Climate change worsens the existing problem of under-nutrition in Africa and will further challenge the current efforts to reduce poverty and under-nutrition [8, 9].

The causes malnutrition include household food insecurity, inadequate care for women and children, and unhealthy environments, poor sanitation and hygiene or lack of health services [10]. As all underlying causes of malnutrition are potentially seasonal, information on seasonal changes in determinants of malnutrition and their effect on linear growth is essential to improve planning and targeting of food security and nutrition-sensitive interventions in agriculture and, ultimately, child well-being [11, 12].

To date, the Demographic and Health Surveys and majority of child growth studies do not consider the seasonal changes when assessing child growth. This hampers assessment of nutritional status of children in many resources limited countries and seasonal priorities. Understanding seasonality of child growth can improve models and simulations to track of success in the fight against malnutrition [13, 14].

The aim of this study was to determine seasonality and determinants of child growth velocity and growth deficit in rural southwest Ethiopia. We hypothesized that (i) children had a higher growth velocity in the post-harvest season than pre-harvest season, (ii) children had a lower child growth deficit in the post-harvest season than pre-harvest season, and (iii) there is a difference in child growth deficit between post and pre-harvest seasons due to seasonal variability of dietary intake, food security, season of child birth and morbidity.

## Methods

### Study design and population

Data for the present study were obtained from four rounds of a longitudinal panel survey conducted in ten districts (woredas) encompassing 20 counties (kebeles) in Oromiya Region and Southern Nations, Nationality and Peoples Region of Ethiopia. Samples of 1200 households were selected using multi-stage cluster sampling of woredas and kebeles. Individual households were sampled at the kebele level using the expanded program on immunization sampling method [15]. Households with a child under 5 years were included in the current analyses (round 1 *n* = 579, round 2 *n* = 674, round 3 *n* = 674 and round 4 *n* = 680). Data were collected using a pre-tested interviewer-administered questionnaire, prepared in Afan Oromo and Amharic, and administered using an electronic tablet. Supervisors transferred data to the central database via a wireless Internet connection using the tablets. Details on the sampling procedure, measurement, construction of aggregate variables, data collection procedures were reported elsewhere [16].

### Seasonality of child growth

According to the Ethiopian National Meteorological Agency, Ethiopia has four agricultural seasons based on the average trends of the weather and rainfall. Summer (lean season) includes 3 months such as June, July and August characterized by heavy rainfalls. Spring (pre-harvest season) includes September, October and November. Winter (harvest season) includes December, January and February. Autumn (post-harvest season) runs from March till and May [17]. In summer (lean season), 97% of all crops and 96% of total cereals are cultivated. The pre-harvest season and post-harvest are typically used as transition phases between the lean and harvest seasons [18, 19].

For the present study, we considered the two main cropping seasons in southwest Ethiopia: the pre-harvest season (September – November) and post-harvest (late February–May) [20].We collected data twice per year during 2 years to assess seasonality of child growth. Data from round one and three were conducted from February 9 till April 9, 2014 and March 4 till May 01, 2015, which was the post-harvest season. Round two and four were conducted from Sept 22 till November 19, 2014 and August 31 till October 29, 2015, which was the pre-harvest season.

### Anthropometric data

A SECA weight scale and length/height boards were used to measure weight and length/height with a precision of 100 g and 1 mm, respectively. Height of children older than 24 months was measured standing while the length of those younger than 24 months was measured in recumbent position as recommended by WHO [21]. The height and weight of caretakers and children were measured without shoes and light clothes [22].To account for differences due to measurement method, 0.7 cm was added to the height values before merging them with the length data [21].

### Growth velocity

Growth velocities were included height and weight velocity. Growth velocity is the change in measurements or increments in weight and length/height from one visit to the next visit. This provides information on growth monitor progress. It indicates the velocity or the rate of growth per unit of time [23].

Before calculating the growth velocity, we constructed Lambda-Mu-Sigma Method (LMS method) which summarizes three curves representing the median (M), the coefficient of variation (S), and the skewness of distribution (L) to pool the age of the child in different rounds [24, 25]. Similarly, length and weight increased much more rapidly in first few months of life compared with the later ages. To address this, age was transformed before smoothing of the centile curve [26]. Growth velocities were calculated as follows: V = M*n + 1* – M*n/Tn + 1-Tn,* M*n* and M*n + 1* were measurements at adjacent occasions, and *Tn + 1-Tn* were the time measurements at adjacent occasions [27].

### Child growth deficits

Child growth deficits were included child linear growth and weight gain. Child growth deficits are representative of physical growth and indicate differences in size over a period of time [21, 23]. We measured child growth deficits (linear growth and weight gain) for longitudinal data using absolute value of height and weight according to WHO recommendation [21].

### Dietary diversity

A child dietary diversity score was calculated from 7 food groups according to the World Health Organization indicators for assessing infant and young child feeding practices [28]: (i) grains and tubers; (ii) milk; (iii) vitamin A-rich fruits/vegetables; (iv) other fruits, vegetables or juices; (v) flesh foods (meat, fish, poultry and liver/organ meats); (vi) egg and (vii) legumes. The household dietary diversity score (HDDS) was calculated from 12 food groups according to the Food and Agriculture Organization [29] and includes (i) cereals; (ii) tubers and roots; (iii) vegetables; (iv) fruits; (v) meat; (vi) eggs; (vii) fish and other seafood; (viii) legumes, nuts and seeds; (ix) milk and milk products; (x) oils and fats; (xi) sweets and (xii) spices, condiments and beverages. Details of the dietary diversity measurement and construction of high, middle and low categories are reported elsewhere [16].

### Household food insecurity

Household food insecurity was measured using the household food insecurity access scale (HFIAS) that was previously validated for use in low-and middle-income countries [30]. The household food insecurity measurement and classification of food secure households, moderately food insecure households and severely food insecurity households were applied to the study area earlier [16].

### Morbidity

Mothers were asked if their child had any illness, diarrhea or a cough in the 2 weeks preceding the data collection. The diagnosis of the three illnesses was based on standardized assessment as used in the Demographic Health Survey questionnaire [31]. Child morbidity was self-reported by mothers.

### Data quality

Before data collection, the questionnaire was pre-tested on 5% of the total sample that was not included in the final main sample. The pre-test was conducted in Yem Special District in SNNP Region and Bedele District in Oromiya region, which has similar characteristics as the main sample. A 12-day intensive training was provided to data collectors and supervisors prior to data collection. The training focused on how to ask questions, their meaning, and how to record the answers. The trainees were also encouraged to ask about issues that are unclear, pay close attention, and take careful notes on issues that they are not familiar. During and after data collection, supervisors monitored the data collection team to ensure their adherence to the study protocol. In addition, the data manager checked all the data submissions from the field on a weekly basis.

### Data analysis

The data were verified for distribution, missing values and outliers, then cleaned and analyzed using STATA version 11 for Windows (STATA Corporation, College Station, TX, USA). We excluded children who had only one observation during the follow-up survey from analysis. Exploratory analyzes were conducted to examine the sample characteristics over the different measurements and rounds. The hierarchical nature of the data was taken into account during the statistical analysis using linear mixed effects model fitted with restricted maximum likelihood estimation method. The models were adjusted for age of the child, seasons of child birth, sex of the child, any illness in the past 2 weeks, child dietary diversity and household food insecurity classification. Multi-collinearity and interaction term were verified in the models. The results are in terms of parameter estimates, standard errors and 95% confidence interval (CI) expressing the findings.

## Result

From the total sample, 579, 674,674 and 680 children under age of 5 years were included in the analysis of round one, two, three and four, respectively. Of the children who were included in the analysis, nine (1.6%) individuals in round two, nine (1.3%) individuals in round three and 17(2.5%) individuals in round four had missing data for all variables.

Overall, 50.8% female and 49.2% of male participated in this study. The overall mean age of the children was 37.2 ± 16.6 months (i.e. round one = 28.8 ± 14.0 months, round two = 34.2 ± 15.7 months, round three = 39.8 ± 15.8 months and round four = 45.1 ± 16.1 months). More than half (55.4%) of the children were under age of 12–36 months.

**Fig. 1:**
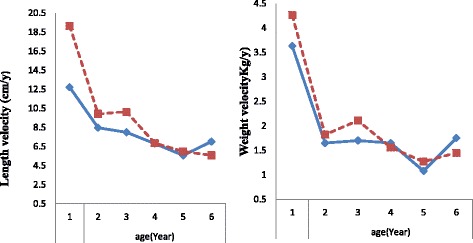
Median length (left) and weight velocity (right) of children in southwest rural Ethiopia by seasons and age, 2014–2015.  Post-harvest season.  Pre-harvest season

Figure 1 shows the median values for height and weight velocity of the children by season and age. A marked decrease in the growth velocity is observed from the first year to the second year of the child. A higher length and weight velocity were observed in pre-harvest season compared with post-harvest season (length velocity = 6.4 cm/year and weight velocity = 0.6 kg/year). Female children showed the highest length velocity in pre-harvest season with an average difference of 4.7 cm/year, while male children had the highest weight velocity in pre-harvest season with an average difference of 0.6 kg/year.

**Fig. 2:**
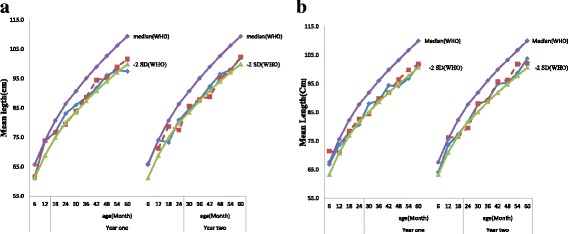
**a** Mean height of female children by year in post and pre harvesting seasons in southwest Ethiopia.  Post-harvest season.  Pre-harvest season. *SD = World Health organization child growth standard reference − 2 standard deviation*. *Median WHO = World Health organization child growth standard reference = 50%*. **b** Mean height of male children by year in post and pre harvesting seasons compared to the WHO reference 2006.  Post-harvest season.  Pre-harvest season. *SD = World Health organization child growth standard reference − 2 standard deviation*. *Median WHO = World Health organization child growth standard reference = 50%*

The growth of almost all children was between WHO median and − 2 z-scores and with a similar growth trend over time. Figure 2a & b shows the seasonal variation in absolute mean length by age and sex of the children. Children had a lower growth deficit compared to the median in the post-harvest season than pre-harvest season. In the pre-harvest season, children had a mean height of 4.3 cm below the heights that corresponded to WHO reference, while post-harvest season children had a height of 5.7 cm below the heights that corresponding to WHO reference. In the pre-harvest season, female and male children had mean heights of 4.7 cm and 4.0 cm below the height corresponding to WHO Median reference respectively. However, this deficit increased to 5.6 cm and 5.7 cm in the post-harvest season for female and male respectively.

Table 1 explains the bivariate association of seasons and exposure variables. Household food insecurity, household dietary diversity, and type of individual food groups consumed (i.e. vitamin A rich vegetables and fruits intake, flesh foods in take, egg intake and legumes intake) were significantly associated with seasonality. Household food insecurity, vitamin-A rich vegetables and fruits, flesh foods (meat, fish, poultry and liver/organ meats) consumption were higher during the pre-harvest season, while household dietary diversity, egg, and legume consumption were higher during the post-harvest season.

**Table 1:** Association between seasons and selected exposure variables in southwest Ethiopia, 2014–15

Variables	Post-harvesting season (*N* = 1253)	Pre-harvesting season (*N* = 1354)	*P* ^*a*^
Household food insecurity, Mean (SD)	5.4 (6.1)	6.8 (6.6)	*0.001*
Household dietary diversity, Mean (SD)	3.9 (1.5)	3.7 (1.4)	*0.001*
Cereal intake, %	48.3	51.7	*0.63*
Vitamin A rich vegetables and fruits intake, %	42.3	57.7	*0.001*
Flesh food intake, %	25.2	74.8	*0.001*
Egg intake, %	63.4	36.6	*0.001*
Dairy intake, %	50.4	49.6	*0.19*
Legume intake, %	51.4	48.6	*0.001*
Other fruit and vegetables intake, %	48.9	51.1	*0.46*
Child dietary diversity score, Mean (SD)	2.9 (1.3)	2.8 (1.2)	*0.77*

^a^Bivariate association was assessed using a Chi-square test

Table 2 shows the association between seasons and child growth deficit (linear growth and weight gain). The absolute mean height of children increased on average 3.3 cm per year in pre-harvest season compared to the post-harvest season. Similarly, the absolute mean weight of children increased by 1.0 kg per year in pre-harvest season compared to the post-harvest season.

**Table 2:** Associations of seasons and child growth deficits over a 2-year follow-up period in Southwest Ethiopia, 2014–15

	Model 1: height		Model 2: weight	
	Estimate (95% CI)	SE	Estimate (95% CI)	SE
Fixed effects				
Intercept	86.93 (86.10, 87.76)**	0.42	11.55 (11.34, 11.75)**	0.10
Seasons				
Post harvest (ref)				
Pre-harvest	3.34 (2.94, 3.73)**	0.20	1.01 (0.91, 1.11)**	0.05
Random-effects				
Variance of random intercept	10.34 (3.94, 4.53)**	0.30	2.54 (2.34, 2.69)**	0.07
Variance of measurement errors (residuals)	5.09 (2.57, 2.90)**	0.08	1.27 (1.23, 1.31)**	0.021

**Significant at *p* < 0.001, *CI* confidence interval

Child linear growth had similar determinants in post and pre-harvest seasons (Table 3). Children with a low dietary diversity and born during the lean season had lower linear growth in both seasons. Age of the child was positively associated with child linear growth in both seasons. Having experienced no illness during the past 2 weeks and severely food insecure household on the other hand was positively associated with child linear growth in post-harvest season.

**Table 3:** Linear growth deficit in the post-and pre-harvesting seasons over a 2-year follow-up period in Southwest Ethiopia, 2014–15

	Model 1 post-harvest		Model 2 pre-harvest	
	Estimate (95% CI)	SE	Estimate (95% CI)	SE
Fixed effects				
Intercept	66.59 (65.51, 67.68)**	0.55	67.79 (66.49, 69.09)**	0.66
Seasons of child birth				
Autumn (ref)				
Spring	0.35 (−0.62, 1.32)	0.50	0.31 (− 0.73, 1.35)	0.53
Summer	−0.99 (−1.93, −.04)*	0.48	−1.06 (−2.07, −.04)*	0.52
Winter	−0.35 (− 1.42, 0.71)	0.54	− 0.25 (− 1.39, 0.89)	0.58
Age of the child (months)	0.60 (0.58, 0.62)**	0.01	0.58 (0.56, 0.60)**	0.01
Sex of the child				
Female (ref)				
Male	0.41 (−0.25, 1.07)	0.34	0.67 (− 0.07, 1.40)	0.38
Any illness in the past 2 weeks				
Yes (ref)				
No	0.54 (0.03, 1.06)*	0.26	0.23 (−0.39, 0.86)	0.32
Child Dietary Diversity				
High (ref)				
Medium	−0.39 (−0.94, 0.15)	0.28	−0.31 (− 0.93, 0.31)	0.32
Low	−1.21 (−1.80, −0.61)**	0.31	−1.44 (−2.12, −0.76)**	0.35
Household food insecurity				
Food secure (ref)				
Moderately food insecure	0.40 (− 0.17, 0.96)	0.29	−0.17 (− 0.84, 0.50)	0.34
Severely food insecure	0.68 (0.06, 1.30)*	0.32	−0.38 (−1.11, 0.36)	0.38
Random-effects				
Variance of random intercept	4.23 (3.94, 4.53)	0.15	4.41 (4.10, 4.75)	0.17
Variance of measurement errors (residuals)	2.73 (2.57, 2.90)	0.09	3.443 (3.26, 3.64)	0.10

*Significant at *p* < 0.05, **Significant at *p* < 0.001, *ref* Reference category, *CI* confidence interval

Factors associated with child weight gain were similar in post and pre-harvest seasons (Table 4). Having a low dietary diversity was negatively associated with child weight gain in both seasons. However, being part of a severely food insecure household was negatively associated with child weight gain in the pre-harvest season. Age of the child, being male and no reported illness experience during the past 2 weeks was positively associated with child weight gain in both seasons.

**Table 4:** Child weight gain in the post and pre harvest seasons over a 2-year follow up period in Southwest Ethiopia, 2014–15

	Model 1 post-harvest		Model 2 pre-harvest	
Fixed effects	Estimate (95% CI)	SE	Estimate (95% CI)	SE
Intercept	6.99 (6.65, 7.33)**	0.170	7.24 (6.88, 7.61)**	0.19
Seasons of child birth				
Autumn (Ref)				
Spring	−0.02 (−0.31, 0.29)	0.15	0.01 (− 0.31, 0.32)	0.16
Summer	−0.35 (−0.64, −0.06)*	0.15	− 0.25 (− 0.56, 0.05)	0.16
Winter	−0.19 (−0.51, 0.14)	0.17	−0.11, (− 0.45, 0.23)	0.18
Age of the child (months)	0.13 (0.13, 0.14)**	0.003	0.14 (0.13, 0.14)**	0.003
Sex of the child				
Female (ref)				
Male	0.44 (0.24, 0.65)**	0.11	0.43 (0.22, 0.65)**	0.11
Any illness in the past 2 weeks				
Yes (ref)				
No	0.20 (0.04, 0.37)*	0.09	0.19 (0.02, 0.35)*	0.08
Child dietary diversity				
High (ref)				
Medium	−0.15 (−0.33, 0.03)	0.09	−0.13 (−0.29, 0.04)	0.08
Low	−0.30 (−0.50, −0.11)**	0.10	− 0.39 (−0.58, −0.21)**	0.09
Household food insecurity				
Food secure (ref)				
Moderately food insecure	0.06 (−0.12, 0.25)	0.09	−0.15 (− 0.33, 0.02)	0.09
Severely food insecure	−0.08 (− 0.28, 0.12)	0.10	− 0.23 (−0.43, −0.03)*	0.10
Random-effects				
Variance of Random Intercept	1.28 (1.18, 1.37)	0.05	1.39 (1.30, 1.48)	0.05
Variance of measurement errors (residuals)	0.91 (0.86, 0.97)	0.03	0.87 (0.83, 0.92)	0.03

**Significant at *p* < 0.001, *significant at *p* < 0.05, *ref* Reference category, *CI* confidence interval

## Discussion

Children in low-and middle-income countries suffer from sub-optimal growth due to seasonality of food production, insufficient dietary intake, food insecurity, morbidity, low use of agricultural technology and poor market access [7–9]. To date however, only a few and mostly outdated studies have addressed seasonality of child growth [13, 14]. This study determined seasonality and determinants of child growth velocity and growth deficit in rural southwest Ethiopia.

In the present study, the child growth velocity sharply decreased between one to 2 years of age. The highest length and weight velocity were observed in the pre-harvest season. This finding is similar to a study conducted in northwestern Iran where a sharp decrease in the velocity growth charts from birth to 2 years of age was observed. These charts have remained relatively stable up to 4 years for both sexes [27].

Similarly to Australian findings [32], the present study showed a higher growth velocity in the pre-harvest season compared to the post-harvest season. In the present study however, the majority of pre-harvest data were collected during a period where some farmers had started to harvest crops. This is not unusual in Ethiopia as the majority of vegetables, fruits and some cereals are harvested early during the harvest season [18, 19].

In addition, the present study estimated that vitamin A-rich vegetables and fruits, meat, fish, poultry and liver/organ meats are consumed more in pre-harvest season than post-harvest season. Contrary to our findings, other studies have shown that child growth velocity was lowest in pre-harvest season. Authors have attributed this to distance to food source, food insecurity, health service utilization and child feeding practice [33–36]. In the present study, the data collection period might not have been totally reflecting the pre-harvest season. Most of the data were collected early during the harvest season during which the most cereals were being harvested.

Female children had a higher length velocity but a lower weight velocity than male children in both seasons. A study from Taiwan showed that female children had lower length velocity than male children [37]. This difference was attributed to gender differences in child feeding, geographical factors. Therefore, appropriate childhood interventions should be considered to prevent childhood obesity and chronic disease development.

This study estimated that children were more likely to increase their height and weight in pre-harvest compared to the post-harvest season. As described earlier, pre-harvest data was partly collected early in the harvest season [18, 19] and children might have had some access to cereals and other crops required for child growth.

Belonging to a highly food insecure household was a significant risk factor for lower child weight gain and a protective factor for increased linear growth in pre-harvest and post-harvest seasons, respectively. Families might have protected children during though shortages of food in the household. During food insecure seasons, families give priority to children and feed them first before the other household members. Previous evidence strongly supports the inverse association of child growth, food insecurity and household dietary diversity [12, 13, 32, 35, 38–40].

Children born during the lean season and with a low dietary diversity had a lower linear growth in post-harvest season compared to the pre-harvest season. Due to seasonal variation in food insecurity and dietary intake in developing countries, the season of childbirth affects linear growth of children. Not only the season of childbirth but also season of preconception and pregnancy is associated with child growth later in life. A study conducted in rural Burkina Faso showed that birth weight, birth length, intrauterine growth retardation, and preterm birth showed significant seasonal variations. Birth weights and birth lengths peaked at the end of the dry season, more precisely in April and May [41].

A study conducted in the UK and Gambia showed that season of birth was associated with birth weight, childhood growth and development, educational attainment and puberty timing in women [42, 43].Therefore, adequate nutrition of the mother and the child should consider seasonality of child growth. The latter can have a profound impact on the child’s growth and development and reduced disease risk, as well as on the protection of maternal health [44]. Undernutrition during pregnancy, affecting fetal growth, is a major determinant of stunting and can lead to consequences such as obesity and nutrition-related non-communicable diseases in adulthood [45].

Age of the child and reporting no illness experience in past 2 weeks was positively associated with linear growth and weight again in post-harvest season and the pre-harvest season. It was also observed that being male had positive effect on weight again in post and pre-harvest seasons. Previous evidence showed that age and sex of the child and illness experience in the past 2 weeks were determinants of weight again and linear growth [33, 38, 46].

Even though dietary diversity was significantly associated with stunting in all age groups, the association of dietary diversity with linear growth was as observed as age of the child increased [28].Therefore, dietary diversity and food frequency should consider the age of the child. Similarly, developing countries should consider seasonality of child growth in designing nutrition interventions to reduce the child growth faltering. Children in such settings are still vulnerable to seasonal food shortages due to rain-fed subsistence farming. Seasonality of food availability increases exposure to food shortages affects health of millions of the poor communities worldwide [3].

The strength of the study was its focus on seasonality of growth. Estimates on seasonality of growth and it determinants among rural southwest Ethiopia can guide planning, implementation and evaluation of integrated promotion of complementary feeding and health seeking behavior and household income generating activities options. Such knowledge can also strengthen partnership between nutrition and agriculture to reduce vulnerability to seasonal food shortages. However, the study did not have data from all four seasons. In addition, we were unable to collect data from the peak of the lean season. A comparison of the lean season and post-harvest season may have shown different and more pronounced results. Increased seasonal nutrition surveillance, which includes all four seasons, should be conducted to understand the seasonality of child growth velocity and deficits.

## Conclusion

The study examined seasonality and determinants of child growth velocity and growth deficit in rural southwest Ethiopia. Child growth velocities were higher in the pre-harvest season than post-harvest season. Children had a higher child growth deficit in the post-harvest season than pre-harvest season corresponding to WHO reference. Child growth deficits had almost similar determinants in post and pre-harvest seasons. Being born during the lean season, a low dietary diversity, belonging to a highly food insecure household and reporting illness experienced during the past 2 weeks were negatively associated with child linear growth and weight gain in rural southwest Ethiopia. Complementary feeding and early health seeking education and household income generating activities options should be design to solve seasonality of child growth velocity and deficit in rural communities in low-and middle-income countries.

## Abbreviations

ENGINE: Empowering New Generations to Improve Nutrition and Economic Opportunities
HDDS: household dietary diversity score
HFIAS: household food insecurity access scale
UK: United Kingdom
UNICEF: United Nations International Children’s Emergency Fund
USAID: United States Agency for International Development
WHO: World Health Organization
